# Supplementary material for: TEM image restoration from fast image streams
Source: PLoS One. 2021 Feb 1;16(2):e0246336. doi: 10.1371/journal.pone.0246336 (PMC7850474; doi:10.1371/journal.pone.0246336)
Supplement: Attachment — Submitted filename: comments on PONE-D-20-33777.pdf [file pone.0246336.s001.pdf]

This manuscript proposed a fast image stream based transmission electron microscopy image restoration by consideration of motion blur and noise. This work itself is interesting. However, some other problems in the manuscript are still concerned in the following:

1. Grammar mistake in the sentence “shows that using deep learning for image restoration of TEM image live streams has great potential but also carry limitations”.
2. The contribution and innovation of this work should be stated more clearly.
3. The organization of this manuscript should be added to the end of the introduction.
4. More details on the method should be exposed in the text.
5. The flow charts of the method should be shown in the manuscript.
6. More evaluation metrics are suggested in the experiments.
7. Could the authors use more kinds of methods in the experiments to validate the methods?
8. More works on image restoration should be included, such as “DOI: 10.1016/bs.mcb.2019.05.001”, “DOI: 10.1109/TGRS.2014.2307354”, “DOI: 10.1111/jmi.12716”…
9. The conclusion is suggested to be simplified.
10. Some references missed the fundamental information, such as volume, number and page. Please check them very carefully.
